# Prevalence and trends of perioperative major adverse cardiovascular and cerebrovascular events during cancer surgeries

**DOI:** 10.1038/s41598-023-29632-7

**Published:** 2023-02-10

**Authors:** Venkataraghavan Ramamoorthy, Kelvin Chan, Sandeep Appunni, Zhenwei Zhang, Md Ashfaq Ahmed, Peter McGranaghan, Anshul Saxena, Muni Rubens

**Affiliations:** 1 Miami Cancer Institute, Baptist Health South Florida, 8900 N Kendall Dr, Miami, FL 33176 USA; 2 Nova Southeastern University, Dr. Kiran C. Patel College of Allopathic Medicine, Davie, FL USA; 3 Government Medical College, Kozhikode, Kerala India; 4 Department of Internal Medicine and Cardiology, Charité—Universitätsmedizin Berlin, Corporate Member of Freie Universität Berlin and Humboldt Universität Zu Berlin, 10117 Berlin, Germany

**Keywords:** Cancer, Cardiology, Medical research

## Abstract

Major adverse cardiovascular and cerebrovascular events (MACCE) is an important cause of morbidity and mortality during perioperative period. In this study, we looked for national trends in perioperative MACCE and its components as well as cancer types associated with high rates of perioperative MACCE during major cancer surgeries. This study was a retrospective analysis of the National Inpatient Sample, 2005–2014. Hospitalizations for surgeries of prostate, bladder, esophagus, pancreas, lung, liver, colorectal, and breast among patients 40 years and greater were included in the analysis. MACCE was defined as a composite measure that included in-hospital all-cause mortality, acute myocardial infarction (AMI), and ischemic stroke. A total of 2,854,810 hospitalizations for major surgeries were included in this study. Of these, 67,316 (2.4%) had perioperative MACCE. Trends of perioperative MACCE showed that it decreased significantly for AMI, death and any MACCE, while stroke did not significantly change during the study period. Logistic regression analysis for perioperative MACCE by cancer types showed that surgeries for esophagus, pancreas, lung, liver, and colorectal cancers had significantly greater odds for perioperative MACCE. The surgeries identified to have greater risks for MACCE in this study could be risk stratified for better informed decision-making and management.

## Introduction

Cancer is the second leading cause of death in the U.S^1^. In 2020 alone, there were approximately 1.8 million newly diagnosed cancer cases and 0.6 million cancer death^2^. Cancer treatments generally included surgery, chemotherapy, and radiotherapy used alone or in conjunction. During the year 2013–2014, 45% of cancer patients decided to choose surgery as their primary cancer treatment option^3^. Cancer surgeries, like other major surgeries, are associated with many complications leading to increased morbidity and mortality. Major adverse cardiovascular and cerebrovascular events (MACCE) constitute an important cause of morbidity and mortality during the perioperative period^4,5^. The risk for prolonged hospitalization and associated expenditures increase significantly due to cardiovascular and cerebrovascular events^6,7^.

During the past few decades, there have been significant advancements in perioperative risk stratification procedures, surgical and anesthetic methods, and efforts to decrease the incidence of MACCE during perioperative period^8–11^. In spite of these efforts, increasing prevalence of cardiovascular risk factors among patients undergoing surgeries dilute these achievements^12^. The implications of these risk factors could be worse for cancer patients receiving surgical treatments due to increased comorbidity burden and treatment side effects. However, there are not many studies that explore the prevalence and trends of perioperative MACCE among patients undergoing cancer surgeries. In a study by Smilowitz et al., MACCE was used to assess perioperative morbidity and mortality after noncardiac surgeries^4^. Although these surgeries could include cancer related surgeries, cancer related estimates were not reported^4^. In the current study, we looked for national trends in prevalence of perioperative MACCE and its components among selected cancer surgeries using a national database. In addition, we also identified the cancer types that were associated with greater risks of perioperative MACCE during surgical treatments.

## Methods

### Study population

The National Inpatient Sample (NIS) from Healthcare Cost and Utilization Project (HCUP) of the U.S. Department of Health & Human Service was used for this study. The NIS database collects approximately 7 million hospital stays each year which equates to 20% of hospitalizations across the whole nation. Patients aged 40 and greater and who were hospitalized for cancer surgeries during 2005 to 2014 were included for the analysis. Patients who received surgeries for primary cancers of prostate, bladder, esophagus, pancreas, lung, liver, colorectal, and breast were included in the analysis. These primary procedures were identified according to their respective International Classification of Diseases, Ninth Revision (ICD-9) code (Supplementary Table 1). Patients’ demographic data including ages undergoing cancer surgery, sex, and race were collected. Patients’ medical history and comorbidities such as obesity, tobacco usage, hypertension, hyperlipidemia, diabetes mellitus, chronic kidney disease, end-stage renal disease, coronary artery disease, prior percutaneous coronary intervention (PCI), prior coronary artery bypass surgery (CABG), peripheral arterial disease, valvular heart disease, history of heart failure, history of venous thromboembolism, chronic pulmonary disease, alcohol abuse, and anemia were identified using ICD-9-CM codes.

### Study outcomes

The primary outcome was to identify potential perioperative MACCE risk factors. MACCE was defined as a composite measure that included in-hospital all-cause mortality, acute myocardial infarction (AMI), and ischemic stroke. Secondary outcome was trends in the prevalence of perioperative MACCE and its individual components during cancer surgeries during the study period. Although MACCE is a less standardized measure of perioperative morbidity and mortality than major adverse cardiovascular events (MACE), MACCE was used because cerebrovascular adverse outcomes are common among cancer patients.

### Statistical analysis

Statistical analyses were performed SAS version 9.4 (SAS Institute, NC), which accounted for the complex survey design and clustering. We followed the AHRQ guidelines for using NIS data to ensure appropriate methods for the study^13^. To improve national estimates NIS was redesigned in 2012. To account for this restructuring, we used trend weight, “TRENDWT”, for the years 2005 to 2011 and regular discharge weight, “DISCWT” for the years 2012 to 2014^14^. We reported frequencies and percentages for categorical variables and mean and standard deviation for continuous variables. Kruskal–Wallis chi-square tests and t tests were used to compare categorical and continuous variables, respectively. We estimated the significance of trends across the study period using Cochran Armitage test for perioperative MACCE and its components. Finally logistic regression analyses were used to identify the association between cancer surgeries and MACCE. The multivariable models were adjusted for factors such as age, race, obesity, tobacco use, hypertension, hyperlipidemia, diabetes mellitus, chronic kidney disease, end-stage renal disease, coronary artery disease, prior revascularization with either percutaneous coronary intervention or coronary artery bypass surgery, peripheral arterial disease, valvular heart disease, congestive heart failure, prior venous thromboembolism, chronic lung disease, alcohol abuse, anemia, and year of hospitalization. All tests were two tailed and statistical significance was set at *P* < 0.05.

## Results

We identified a total of 2,854,810 hospitalizations for major surgeries among patients ≥ 40 years of age during 2005–2014 (Supplementary Fig. 1). Among these 67,316 (2.4%) had, while 2,787,494 (97.6%) did not have perioperative MACCE. Demographic and clinical characteristics of patients undergoing cancer surgery with and without perioperative MACCE is shown in Table 1.

**Table 1:** Characteristics of patients undergoing cancer surgery with and without perioperative major adverse cardiovascular and cerebrovascular events.

Characteristic	All surgeries (n = 2,854,810)	Perioperative MACCE (n = 67,316, 2.4%)	No perioperative MACCE (n = 2,787,494, 97.6%)	*P* value
Age in years, mean (SE)	65.4 (0.07)	74.0 (0.11)	65.2 (0.07)	< 0.001
Female, n (%)	1,304,362 (45.8%)	28,023 (41.6%)	1,276,339 (45.9%)	< 0.001
Race, n (%)				< 0.001
White	1,871,516 (65.6%)	45,769 (68.0%)	1,825,747 (65.5%)	
Black	248,431 (8.7%)	5390 (8.0%)	243,040 (8.7%)	
Hispanic	149,785 (5.2%)	2914 (4.3%)	146,870 (5.3%)	
Other race	139,627 (4.9%)	2925 (4.3%)	136,703 (4.9%)	
Unknown	445,451 (15.6%)	10,318 (15.3%)	435,133 (15.6%)	
Obesity, n (%)	226,148 (7.9%)	3798 (5.6%)	222,350 (8.0%)	< 0.001
Tobacco use, n (%)	770,876 (27%)	13,627 (20.2%)	757,249 (27.2%)	< 0.001
Hypertension, n (%)	1,490,484 (52.2%)	36,143 (53.7%)	1,454,342 (52.2%)	< 0.001
Hyperlipidemia, n (%)	801,767 (28.1%)	16,502 (24.5%)	785,265 (28.2%)	< 0.001
Diabetes mellitus, n (%)	515,658 (18.1%)	15,319 (22.8%)	500,339 (17.9%)	< 0.001
Chronic kidney disease, n (%)	101,353 (3.6%)	7631 (11.3%)	93,722 (3.4%)	< 0.001
End-stage renal disease, n (%)	12,346 (0.4%)	1254 (1.9%)	11,092 (0.4%)	< 0.001
Coronary artery disease, n (%)	400,063 (14%)	31,423 (46.7%)	368,641 (13.2%)	< 0.001
Prior PCI, n (%)	99,833 (3.5%)	3510 (5.2%)	96,323 (3.5%)	< 0.001
Prior CABG, n (%)	95,867 (3.4%)	3402 (5.1%)	92,465 (3.3%)	< 0.001
Peripheral arterial disease, n (%)	82,403 (2.9%)	6840 (10.2%)	75,563 (2.7%)	< 0.001
Valvular heart disease, n (%)	108,121 (3.8%)	5621 (8.4%)	102,500 (3.7%)	< 0.001
History of heart failure, n (%)	18,469 (0.6%)	1714 (2.5%)	16,755 (0.6%)	< 0.001
History of venous thromboembolism, n (%)	50,918 (1.8%)	1078 (1.6%)	49,839 (1.8%)	< 0.001
Chronic pulmonary disease, n (%)	510,830 (17.9%)	19,604 (29.1%)	491,226 (17.6%)	< 0.001
Alcohol abuse, n (%)	49,040 (1.7%)	1953 (2.9%)	47,086 (1.7%)	< 0.001
Anemia, n (%)	445,889 (15.6%)	18,451 (27.4%)	427,438 (15.3%)	< 0.001
Elective surgery, n (%)	2,363,041 (82.9%)	37,068 (55.2%)	2,325,973 (83.6%)	< 0.001
Cancer surgery type, n (%)				< 0.001
Prostate	710,133 (24.9%)	1729 (2.6%)	708,404 (25.4%)	
Bladder	94,323 (3.3%)	3341 (5.0%)	90,982 (3.3%)	
Esophagus	20,593 (0.70%)	1358 (2.0%)	19,235 (0.7%)	
Pancreas	71,945 (2.5%)	3438 (5.1%)	68,507 (2.5%)	
Lung	391,763 (13.7%)	14,052 (20.9%)	377,711 (13.6%)	
Liver	37,744 (1.3%)	1974 (2.9%)	35,770 (1.3%)	
Colorectal	984,114 (34.5%)	39,615 (58.8%)	944,499 (33.9%)	
Breast	544,195 (19.1%)	1810 (2.7%)	542,385 (19.5%)	

*CABG* coronary artery bypass surgery, *MACCE* major adverse cardiovascular and cerebrovascular events, *PCI* percutaneous coronary intervention.

**Figure 1:**
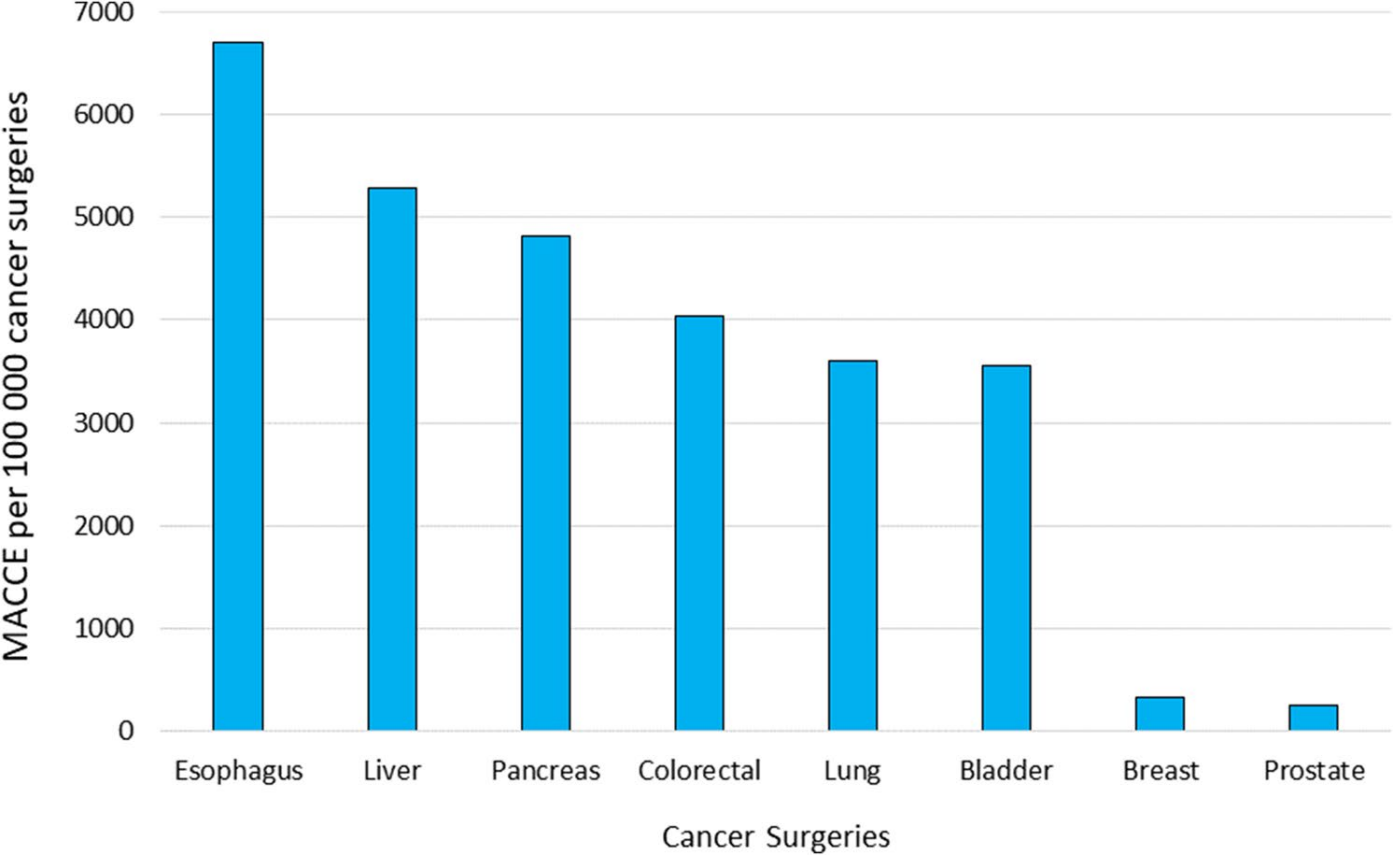
Frequency of perioperative MACCE by type of cancer surgery.

The prevalence of perioperative MACCE was the highest among surgeries for esophageal cancers (6706 per 100,000 cancer surgeries), followed by liver (5284 per 100,000 cancer surgeries), pancreatic (4820 per 100,000 cancer surgeries), colorectal (4038 per 100,000 cancer surgeries), lung (3599 per 100,000 cancer surgeries), bladder (3560 per 100,000 cancer surgeries), breast (332 per 100,000 cancer surgeries) and prostate (246 per 100,000 cancer surgeries) cancers (Fig. 1).

**Figure 2:**
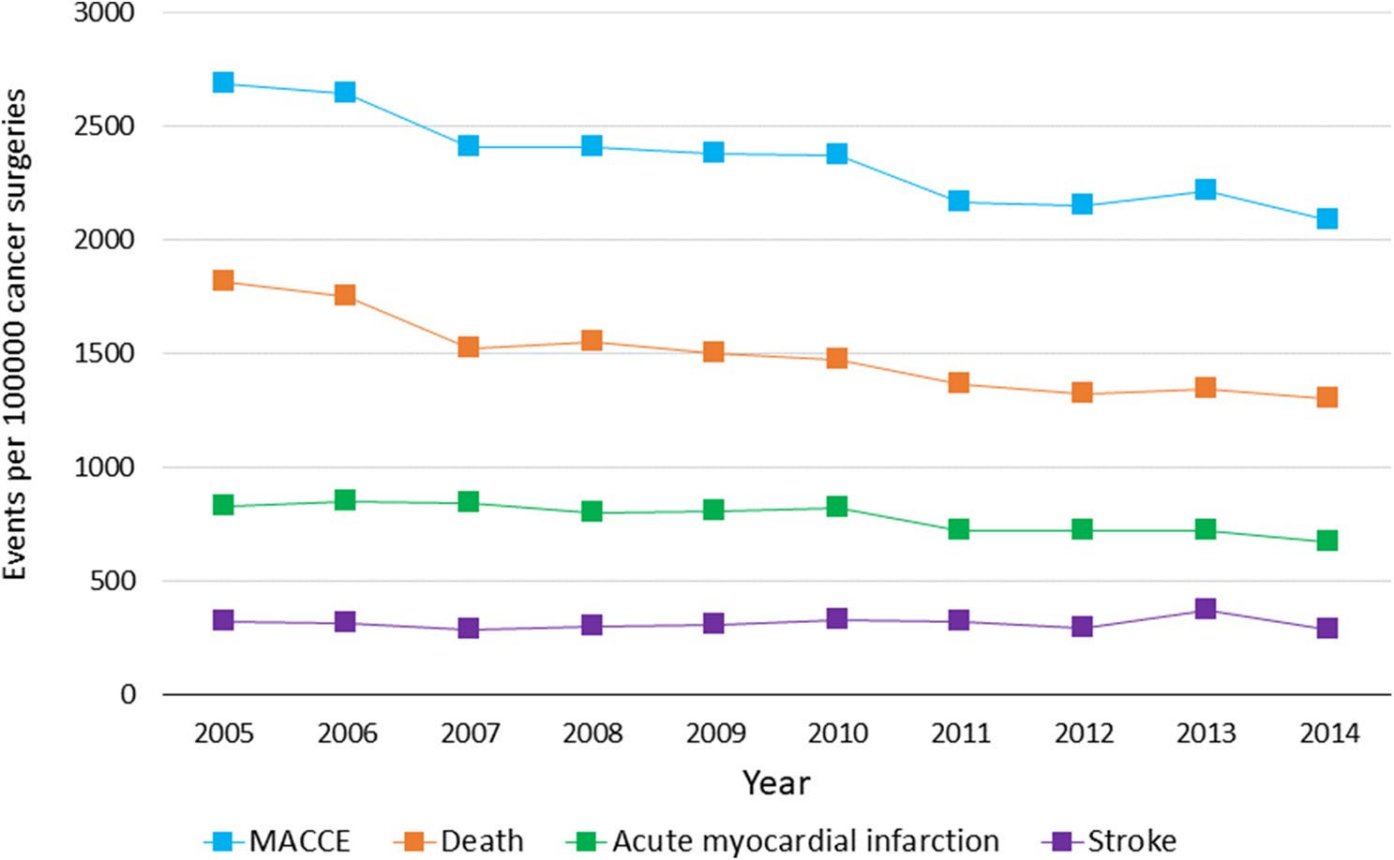
Trends in prevalence of perioperative MACCE over time.

Trends in prevalence of perioperative MACCE showed that AMI (relative decrease, − 27.6, *P* for trend 0.002), death (relative decrease, − 35.2%, *P* for trend < 0.001), and any MACEE (relative decrease, − 29.4%, *P* for trend < 0.001) significantly decrease during the study period, while the prevalence of stroke decreased, though not significantly (relative decrease, − 8.4%, *P* for trend 0.608) (Table 2). Figure 2 shows trends in prevalence of perioperative MACCE and its components over the study period.

**Table 2:** Trends in cardiovascular outcomes of major cancer surgery over time.

Characteristic	2005–2006 (n = 570,159)	2007–2008 (n = 633,705)	2009–2010 (n = 583,819)	2011–2012 (n = 568,057)	2013–2014 (n = 499,070)	*P* value
Age in years, mean (SE)	66.1 (0.10)	65.3 (0.16)	65.3 (0.13)	65.2 (0.10)	65.4 (0.06)	< 0.001
Female, n (%)	279,347 (49.1%)	289,083 (45.7%)	264,014 (45.3%)	253,593 (44.7%)	218,325 (43.7%)	< 0.001
Cancer surgery type, n (%)						< 0.001
Prostate	124,162 (21.8%)	170,106 (26.8%)	151,840 (26%)	146,970 (25.9%)	117,055 (23.5%)	
Bladder	16,004 (2.8%)	19,324 (3%)	19,492 (3.3%)	19,872 (3.5%)	19,630 (3.9%)	
Esophagus	3689 (0.6%)	3982 (0.6%)	4327 (0.7%)	4256 (0.7%)	4340 (0.9%)	
Pancreas	9835 (1.7%)	13,696 (2.2%)	15,336 (2.6%)	16,734 (2.9%)	16,345 (3.3%)	
Lung	86,039 (15.1%)	85,784 (13.5%)	76,427 (13.1%)	72,748 (12.8%)	70,765 (14.2%)	
Liver	5303 (0.9%)	7798 (1.2%)	8189 (1.4%)	7964 (1.4%)	8490 (1.7%)	
Colorectal	202,869 (35.6%)	204,730 (32.3%)	195,152 (33.4%)	194,667 (34.3%)	186,695 (37.4%)	
Breast	122,258 (21.4%)	128,285 (20.2%)	113,056 (19.4%)	104,846 (18.5%)	75,750 (15.2%)	
Major adverse cardiovascular events						
Any MACCE	15,198 (2.7%)	15,264 (2.4%)	13,871 (2.4%)	12,254 (2.2%)	10,730 (2.2%)	< 0.001
Death	10,156 (1.8%)	9725 (1.5%)	8673 (1.5%)	7620 (1.3%)	6580 (1.3%)	< 0.001
AMI	4782 (0.8%)	5198 (0.8%)	4746 (0.8%)	4098 (0.7%)	3460 (0.7%)	0.002
Stroke	1790 (0.3%)	1842 (0.3%)	1842 (0.3%)	1726 (0.3%)	1640 (0.3%)	0.608

*AMI* acute myocardial infarction, *MACCE* major adverse cardiovascular and cerebrovascular events.

Logistic regression analysis for perioperative MACCE by cancer types showed that surgeries for esophagus (OR, 7.89 (6.88–9.04), pancreas (OR, 5.76 95% CI: 4.33, 6.87), lung (OR, 6.70 95% CI: 5.98–7.52), liver (OR, 4.51 95% CI: 2.21–7.25), and colorectal (OR, 3.21 95% CI: 2.14–5.42) cancers had significantly greater odds while surgeries for prostate (OR, 0.94 95% CI: 0.81–0.97) and breast (OR, 0.79 95% CI 0.56–0.82) cancers had significantly lower odds for perioperative MACCE (Table 3).

**Table 3:** Association of cancer surgeries and perioperative major adverse cardiovascular and cerebrovascular events.

Type of surgery	Adjusted odds ratio (95% CI)^a^
	MACCE
Cancer surgery type, n (%)	
Prostate	0.94 (0.81, 0.97)
Bladder	1.68 (0.81, 1.97)
Esophagus	7.89 (6.88, 9.04)
Pancreas	5.76 (4.33, 6.87)
Lung	6.7 (5.98, 7.52)
Liver	4.51 (2.21, 7.25)
Colorectal	3.21 (2.14, 5.42)
Breast	0.79 (0.56, 0.82)

*MACCE* major adverse cardiovascular and cerebrovascular events.

^a^Multivariable models include age, race, obesity, tobacco use, hypertension, hyperlipidemia, diabetes mellitus, chronic kidney disease, end-stage renal disease, coronary artery disease, prior revascularization with either percutaneous coronary intervention or coronary artery bypass surgery, peripheral arterial disease, valvular heart disease, congestive heart failure, prior venous thromboembolism, chronic lung disease, alcohol abuse, anemia, and year of hospitalization as covariates.

## Discussion

Using a national database with 2.8 million major cancer surgeries, our study showed that the prevalence of perioperative MACCE was 2.4% and surgeries for esophageal, lung, liver, colorectal, and pancreatic cancers carried the greatest risks. Trends of perioperative MACCE showed that it decreased significantly for AMI, death and any MACCE during the study period.

Ever since the development of multifactorial index of cardiac risk by Goldman and colleagues, adverse cardiovascular events have been recognized a major cause of morbidity and mortality, especially during the perioperative period^8^. However, since the last four decades there have been ongoing efforts to promptly identify the patients who are at greatest risk of experiencing perioperative MACCE and thereby decrease the risk of morbidity and mortality associated with cancer surgeries. Our analysis showed that the prevalence of perioperative MACCE is still as high as 1 in every 42 surgeries for cancers. To the best of our knowledge, ours is the first study that provides national estimates on perioperative MACCE among cancer surgeries using multivariable regression models adjusting for a wide variety of covariates. Our study is also one of the largest nationally representative trend analyses given the substantially greater number of hospitalizations included for the analysis.

We observed a generalized reduction in perioperative MACCE as well as its components such as death and AMI which is highly encouraging. This could be due to significant improvement in factors associated with surgeries such as improved protocols for surgical case selection, preoperative identification, management, and control of cardiovascular.

risk factors and diseases, improved logistics for minimally invasive surgeries such as microscopic, laparoscopic, and robotic techniques, improved surgical methodologies and expertise, better anesthetic methods, and improved intraoperative and postoperative supervision. Our finding of decrease in AMI is also very reassuring given the fact that there have been significant improvements in diagnostic methods for identifying this condition during the time span of this study^15^. Substantial improvements in prevention measures, pharmacological management and procedural methods for preventing and treating coronary artery disease could be responsible for this finding^16–18^. Findings similar to ours was observed in a study by Smilowitz et al., although it was primarily among non-cardiac surgeries^4^.

We found that there was no significant decline in perioperative stroke rates during the study period. This is concerning given the fact that the incidence of stroke has substantially decreased among US population during the study period^19,20^. Increase in neurological risk factors, atherosclerotic changes, arrhythmias, carotid artery stenosis, cerebrovascular diseases, and deficit in hemodynamic stability during surgeries could be responsible for these findings^21^. In addition, corrective medications for achieving hemodynamic stability such as beta blockers could be responsible for precipitating intraoperative strokes^10^. The lack of decline in perioperative stroke found in our study could also be due to the fact that cancer patients are at increased risk for thromboembolic events, ischemic complications, and vascular occlusion due to common risk factors such as smoking and diabetes mellitus, and shared pathophysiology of increased inflammation^22^. In addition, the complications of cancer treatment and management could also significantly increase the risk for atherosclerosis, leading to stroke.^23^ We also found that perioperative stroke and surgery for lung cancer were strongly associated. This could be due to higher rates of common risk factors such as smoking and obesity^24^. This relation was significant even after excluding patients who received prior treatments for stroke indicating the concerning levels of associations between perioperative stroke and cancer surgeries.

## Limitations

In spite of these findings, our study has some limitations. We used MACCE which is a less standardized measure of morbidity and mortality during perioperative period, instead of MACE. MACCE does not include transient ischemic attack (TIA), though it contains stroke. We used MACCE because cerebrovascular adverse outcomes are common among cancer patients. However, these limitations could have led to some misclassification bias. Another limitation of using MACCE in our study is that it only includes in-hospital mortality. Over the last several years hospital length of stay for surgery has decreased significantly. The decreased rate of MACCE over the study period could be due to decrease in in-hospital mortality over time due to decreased hospital length of stay. We used NIS database for our study. NIS being an administrative database, extracted data is susceptible to coding errors and misclassification bias. This could have adversely affected some of our estimations. Our sample included only hospitalizations for cancer surgeries among adults ≥ 40 years of age because this population is at greater risk of having cardiovascular risk factors. Therefore, our findings are not applicable to younger patients receiving surgeries for cancers. NIS being an administrative database, it does not have information on grading and staging of cancers or the exact timing of AMI or stroke. Availability of such information could have substantially improved our findings. However, we can assure that cancer patients presenting as index admissions for AMI or stroke are much less likely to undergo surgical treatments during those admissions. Therefore, we could assure that AMI and stoke that have been reported in this study are more likely to be perioperative. NIS does not have information on medications administered during the hospitalizations for surgeries. Therefore, we could not have valuable information on anticoagulants, antiplatelets, and beta blockers used during the surgeries or perioperative period. Though NIS has prior heart failure as a variable, it does not have information on echocardiography or biomarkers such as BNP to assess the functioning of ventricles. Although data beyond 2014 were available, we restricted the analysis until 2014 to avoid issues related to ICD-9 to ICD-10 crosswalk. Finally, NIS does not have information on myocardial injury after surgeries. Availability of this information could have substantially improved our ability to estimate the effect of myocardial injury on long term morbidity and mortality among our cohort of patients.

## Conclusions

To the best of our knowledge, this is the first study that explored the prevalence and trends of MACCE among patients receiving surgical treatments for cancers. We found that surgeries for esophageal, lung, liver, colorectal, and pancreatic cancers were associated with significantly greater risk for perioperative MACCE. The prevalence of perioperative MACCE during cancer surgeries showed significantly decreasing trends during 2005 to 2014. The prevalence of components of MACCE such as AMI and death showed significantly decreasing trends while stroke showed decreasing but non-significant trend. Though there was significant decrease in the generalized prevalence of MACCE in cancer surgeries, the lack of expected levels of decrease in stroke should be explored in future studies. The surgeries identified to have greater risks for MACCE in this study could be risk stratified using predictive models. This could help in informed decision-making process for these surgeries to improve hospital outcomes.

## Supplementary Information

Supplementary Information.

## Data Availability

The datasets analyzed during the current study are available in the National Inpatient Sample repository, link: https://www.hcup-us.ahrq.gov/db/nation/nis/nisdbdocumentation.jsp.
