# Supplementary material for: Prevalence and trends of perioperative major adverse cardiovascular and cerebrovascular events during cancer surgeries
Source: Sci Rep. 2023 Feb 10;13:2410. doi: 10.1038/s41598-023-29632-7 (PMC9918731; doi:10.1038/s41598-023-29632-7)
Supplement: Supplementary file 1 — Supplementary Information. [file 41598_2023_29632_MOESM1_ESM.docx]

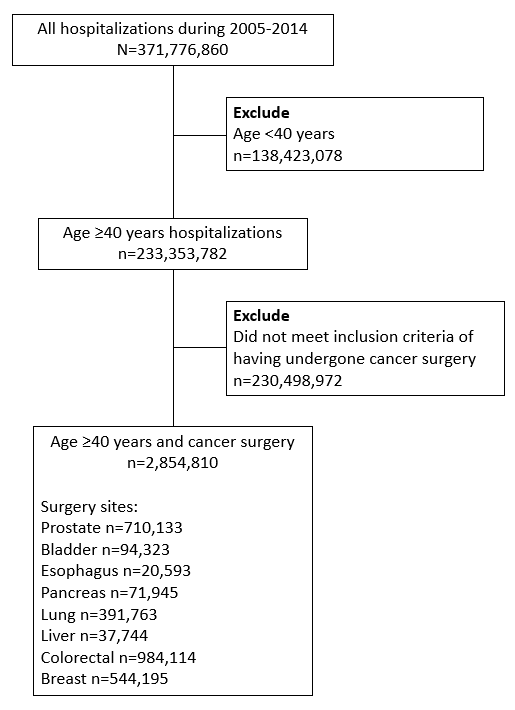


Supplementary Figure 1. Flow diagram describing the inclusion criteria for the study

Supplementary Table 1. ICD-9 diagnosis and procedure codes and CCS code

| **Condition** | **Codes** |
| --- | --- |
| Acute myocardial infarction | CCS code: 100 |
| Acute ischemic stroke | ICD-9 diagnosis code: 433.x1, 434.x1, 436, and 437.1 |
| Complete heart block | ICD-9 diagnosis code: 426.0 |
| Cardiogenic shock | ICD-9 diagnosis code: 785.51 |
| Cardiac arrest | ICD-9 diagnosis code: 427.5 |
|  |  |
| Surgery for prostate cancer | ICD-9 diagnosis codes: 185  And  ICD-9 Procedure Codes: 60.3, 60.4, 60.5, 60.6, 60.61, 60.62, 60.69 |
| Surgery for bladder cancer | ICD-9 diagnosis codes: 188, 188.0-188.9  And  ICD-9 Procedure Codes: 57.6, 57.7, 57.71, 57.79 |
| Surgery for esophagus cancer | ICD-9 diagnosis codes: 150, 150.0-150.9  And  ICD-9 Procedure Codes: 42.04, 42.40, 42.41, 42.42 |
| Surgery for pancreas cancer | ICD-9 diagnosis codes: 157, 157.0-157.9  And  ICD-9 Procedure Codes: 52.2, 52.22, 52.52, 52.51, 52.7, 52.53, 52.6, 52.5, 52.59 |
| Surgery for lung cancer | ICD-9 diagnosis codes: 162, 162.0-162.9  And  ICD-9 Procedure Codes: 32.3, 32.9, 32.29, 32.3, 32.4, 32.5 |
| Surgery for liver cancer | ICD-9 diagnosis codes: 155, 155.0-155.2  And  ICD-9 Procedure Codes: 50.2, 50.22, 50.29, 50.3, 50.4 |
| Surgery for colorectal cancer | ICD-9 diagnosis codes: 153.x, 154.0-154.3, 154.8  And  ICD-9 diagnosis codes: 17.33-17.36, 17.39, 45.7x, 45.80-45.82, 48.42-48.43, 48.49, 48.50-48.52, 48.63-48.65 |
| Surgery for breast cancer | ICD-9 diagnosis codes: 174.x  And  ICD-9 Procedure Codes: 85.41-85.48, 85.20-85.23 |
|  |  |
| Tobacco use | ICD-9 diagnosis codes: 305.1, V15.82 |
| Hyperlipidemia | CCS code: 53 |
| Chronic kidney disease | ICD-9 diagnosis codes: 403.11, 404.12, 404.13, 404.92, 404.92, 585, 585.1, 585.2, 585.3, 585.4, 585.5, 585.9, 586, 587 |
| End-stage renal disease | ICD-9 diagnosis codes: 585.6, 585 with V45.1 |
| Prior PCI | ICD-9 diagnosis codes: V45.82 |
| Prior CABG | ICD-9 diagnosis codes: V45.81 |
| Peripheral arterial disease | ICD-9 diagnosis codes: 440.0, 440.1, 440.2, 440.20, 440.21, 440.22, 440.23, 440.29, 440.4, 440.8, 440.9, 443.9, 557.0, 557.1, 557.9 |
| History of venous thromboembolism | ICD-9 diagnosis codes: V12.51 |
| Anemia | CCS code: 59 |
| Ischemic heart disease | ICD-9 diagnosis code: 410.xx-414.xx |
| Heart failure | ICD-9 diagnosis code: 276.6, 398.91, 402.01, 402.11, 402.91, 404.01, 404.03, 404.11, 404.13, 404.91, 404.93, 425, 428 |
| Prior transient ischemic attack or stroke | ICD-9 diagnosis code: V12.54 |
